# Concerns about Breast Cancer, Pain, and Fatigue in Non-Metastatic Breast Cancer Patients Undergoing Primary Treatment

**DOI:** 10.3390/healthcare4030062

**Published:** 2016-08-26

**Authors:** Chelsea R. Amiel, Hannah M. Fisher, Michael H. Antoni

**Affiliations:** 1 Department of Psychology, University of Miami, Coral Gables, FL 33124, USA; mantoni@miami.edu; 2 Sylvester Cancer Center, Miller School of Medicine, University of Miami, Miami, FL 33136, USA

**Keywords:** breast cancer, depression, social support, rejection, fatigue, pain, survivorship

## Abstract

Women diagnosed with breast cancer often endorse psychosocial concerns prior to treatment, which may influence symptom experiences. Among these, low perceived social support relates to elevated fatigue. Those with low social support perceptions may also experience a greater sense of rejection. We sought to determine if social rejection concerns post-surgery predict fatigue interference 12 months later in women with non-metastatic breast cancer. Depressive symptoms and pain severity after completion of adjuvant therapy (six months post-surgery) were examined as potential mediators. Women (N = 240) with non-metastatic breast cancer were recruited 2–10 weeks post-surgery. Multiple regression analyses examined relationships among variables adjusting for relevant covariates. Greater rejection concerns at study entry predicted greater fatigue interference 12 months later (*p* < 0.01). Pain severity after adjuvant therapy partially mediated the relationship between social rejection concerns and fatigue interference, with significant indirect (*β* = 0.06, 95% CI (0.009, 0.176)) and direct effects (*β* = 0.18, SE = 0.07, *t*(146) = 2.78, *p* < 0.01, 95% CI (0.053, 0.311)). Therefore, pain levels post-treatment may affect how concerns of social rejection relate to subsequent fatigue interference. Interventions targeting fears of social rejection and interpersonal skills early in treatment may reduce physical symptom burden during treatment and into survivorship.

## 1. Introduction

As one of the most common cancers in the USA, breast cancer is the second leading cause of death among American women [1]. Throughout the breast cancer experience, women report poor psychological well-being and disabling physical symptoms that often interfere with daily functioning [2,3,4,5].

Patients with newly diagnosed breast cancer endorse many concerns pertaining to their diagnosis and treatment (e.g., recurrence, death, changes to body image, financial burden etc.) [5]. Moreover, women often express concern regarding how their illness will affect and be accepted by those around them [6]. As such, fear of social rejection is a particularly salient concern endorsed by women recently diagnosed with breast cancer [7]. In fact, Meyerowitz (1980) theorized that perceived accessibility of supportive social relationships is likely to influence the degree of adjustment to a breast cancer diagnosis [2]. If women fear social rejection due to perceived stigma related to breast cancer and its treatment, they may become withdrawn and avoid opportunities for social support [8]. This is disadvantageous as many studies have shown that greater perceived social support predicts better psychological and physical adjustment to breast cancer [9,10,11,12].

It is well known that the period of time surrounding a breast cancer diagnosis is particularly stressful yet women continue to experience distressing psychological and physical sequelae throughout the entire course of treatment. It is common for women with breast cancer to experience symptoms of depression during treatment as they adjust to their diagnosis, loss of breast tissue, and side effects of adjuvant therapies [3,13,14,15,16]. Additionally, the treatment course for breast cancer is often characterized by considerable pain [17,18,19,20], with 25% to 60% of women developing significant pain after surgery and treatment [21,22].

Research has also examined factors related to individual differences in symptom experiences throughout breast cancer survivorship. Cancer-related fatigue (defined as tiredness and a lack of energy that interferes with patients’ quality of life), is one of the most frequently reported symptoms by women with breast cancer [23,24,25]. Although cancer-related fatigue can be present at any time during treatment, extant literature demonstrates that it is most commonly reported after adjuvant treatment and throughout survivorship [25]. In a large sample of breast cancer survivors who were all post-treatment, more than 30% of the women endorsed significant chronic fatigue [26]. Similarly, in another study of cancer survivors, many of whom were breast cancer survivors, 37% reported significant cancer-related fatigue post-treatment [27]. The depression, pain, and fatigue symptom cluster associated with chronic illnesses such as breast cancer is well-established [28], thus a growing body of research has turned its focus towards elucidating the temporal relationships between these psychological and physical symptoms.

Although no studies to our knowledge have examined the relationship between depressive symptoms and fear of social rejection specifically, Eom and colleagues demonstrated that a mixed sample of cancer patients with low perceived social support at the time of diagnosis exhibited higher levels of subsequent depression [29]. Similarly, Hughes et al. (2001) found that low social support prior to treatment was associated with greater depressive symptoms after completion of breast cancer treatment [30]. Loneliness at the time of diagnosis also predicts elevated depressive symptoms in the months following treatment [31].

Importantly, both perceptions of social support at the time of diagnosis and depressive symptoms during breast cancer treatment have been linked to cancer-related fatigue well in to survivorship [26,28,32,33,34]. Geinitz et al. (2004) demonstrated that breast cancer patients’ pre-treatment levels of depressive symptoms predicted elevated levels of fatigue after completion of breast cancer treatment [35]. With existing support for the relationship between low perceived social support and depressive symptoms, and between depressive symptoms and fatigue, it is plausible that concerns about social rejection may relate to fatigue during survivorship indirectly via increased depressive symptoms.

Extant literature has also shown that low perceived social support is linked to higher levels of pain [28,29,30] and fatigue [28,33,34] in cancer patients. Specifically, low social support and loneliness prior to treatment is linked to greater reported pain after breast cancer treatment [30]. Likewise, perceived loneliness is related to greater cancer-related fatigue after completion of adjuvant therapy for breast cancer [28,33,34]. Because of these previous findings, we hypothesize that fears of social rejection would also predict pain and fatigue levels.

Based on this, it is relevant to consider whether pain experiences during treatment predict greater fatigue symptomology after treatment is completed. Blesch and colleagues demonstrated that level of pain was a significant correlate of persistent fatigue in the months following breast cancer treatment [36]. This finding is further supported by Nieboer et al., who found that patients with fatigue had significantly more muscle and joint pain during treatment for breast cancer [37]. Taken together, these findings suggest that the relationship between psychosocial processes and persisting fatigue after completion of adjuvant therapy may be mediated by pain during breast cancer treatment.

The present study will focus on concerns related to fears of rejection and social isolation just after surgery as independent predictors of psychological and physical functioning throughout the breast cancer experience. The study specifically aims to determine the longitudinal relationships between post-surgical rejection concerns and the symptom cluster of depression, pain, and fatigue that is commonly observed throughout breast cancer treatment and survivorship. To our knowledge, this is the first report to examine these relationships in a breast cancer population, and therefore our aims are more exploratory in nature. We did so with a longitudinal study design covering three points in time. First, we examined whether rejection concerns shortly after surgery for breast cancer (prior to the beginning of adjuvant therapy) predicted subsequent fatigue disruption 12 months later. Then, we examined whether depressive symptoms and pain severity just after adjuvant therapy had been completed (six months after study-entry) mediated the association between post-surgery rejection concerns and 12-month fatigue experiences.

## 2. Materials and Methods

### 2.1. Participants

Participants were 240 women with Stage 0-IIIb breast cancer who were 2–10 weeks post-surgery (lumpectomy and mastectomy) and enrolled in a randomized controlled trial between 1998 and 2005. The trial tested the effects of a psychosocial intervention, Cognitive Behavioral Stress Management (CBSM). The study was a single center, single blind, randomized, parallel assignment efficacy trial approved by the Human Subjects Research Office of the University of Miami (UM) Institutional Review Board (IRB; National Institutes of Health Clinical Trial NCT01422551). A detailed description of the original study design is available in previous reports [38,39]. Exclusion criteria included: (1) a diagnosis of stage IV breast cancer or prior cancer (except minor skin cancers such as squamous or basal cell carcinomas); (2) ongoing neoadjuvant or post-surgical adjuvant treatment; (3) a major medical condition other than cancer; (4) falling outside the age range of 21–75 years of age; (5) non-fluency in English; (6) previous hospitalization for psychiatric conditions; and (7) current psychosis, suicidality, major depressive disorder or panic disorder.

### 2.2. Procedures

From a total screening sample of 502 women, 240 were consented, enrolled, completed a baseline assessment, and were then randomized to CBSM intervention or a 1-day psychoeducational control group. Randomization and assessments were completed by blinded study coordinators. Assessments were initially conducted at study-entry, 6-months and 12-months post-study enrollment.

### 2.3. Measures

#### 2.3.1. Demographic and Medical Characteristics

At the time of enrollment, information was collected regarding demographic (e.g., age, race/ethnicity, partnered status) and socioeconomic (e.g., income) status by self-report. Medical information was first collected by self-report and was then verified with medical chart review. Measures of medical status included stage of disease, surgical procedure, and use of prescription medication for pain, anxiety, depression, and sleep.

#### 2.3.2. Psychosocial Measures

Pain severity. The 4-item Pain Severity subscale of the Brief Pain Inventory (BPI) was used to assess participants’ current pain [40]. Women were asked to rate (1) their worst pain intensity during the past day; (2) their least pain intensity during the past day; (3) their average pain intensity during the past day; and (4) their current pain intensity, on a 1 = no pain to 9 = worst pain imaginable scale. These ratings were averaged to obtain a pain severity score, ranging from 1 to 9, with higher scores indicating greater pain severity. The BPI has demonstrated adequate reliability and sensitivity in prior studies investigating pain severity in cancer patients [41,42]. Reliability in the present study was high (α = 0.96).

Depressive symptoms. The presence and severity of depressive symptoms (i.e., mood, guilt, suicide ideation, insomnia, psychomotor agitation or retardation, anxiety, weight loss, and somatic symptoms) over the past week was measured using the 17-item interview-based Hamilton Rating Scale-Depression (HRSD) [43]. A clinical psychologist with extensive training in use of the HRSD trained study assessors based on the structured interview guide [44]. Magnitudes of depressive symptoms were considered on a continuum ranging from 0 to 23, with higher scores indicating greater depressive symptomology. High interrater reliability, internal consistency, and discriminant validity have been confirmed for this scale previously [43]. The HRSD has previously been used in samples of women with breast cancer [45,46]. Internal consistency for the current sample was adequate (α = 0.79).

Rejection issues. Participants’ concerns regarding social rejection due to their breast cancer and/or treatment was measured using the 3-item Rejection Issues subscale of the 28-item Profile of Concerns about Breast Cancer (PCBC) [5]. This measure lists specific concerns relating to breast cancer diagnosis and/or medical treatment and asks the respondent to indicate how concerned she was about each of these over the last few days. Response options range from 1 = not at all concerned to 5 = extremely concerned. The Rejection Issues subscale of the PCBC includes 3 items (i.e., “As you think about your illness, how much are you concerned that your family will become angry with you,” “As you think about your illness, how much are you concerned that your friends will avoid you,” and “As you think about your illness, how much are you concerned that your friends will act as though your disease is contagious”). Ratings for these rejection concerns were summed to obtain a total score for concerns with Rejection issues. Scores range from 3 to 15, with higher scores indicating greater rejection issues. The PCBC has been previously used in samples of women with breast cancer [5]. Cronbach’s alpha for the Rejection Issues subscale was adequate (α = 0.80).

Fatigue interference. Fatigue interference was assessed using the 6-item Perceived Interference subscale of the Fatigue Symptom Index (FSI-I), which was developed for and validated in cancer patients [47,48]. Using a 11-point scale with 0 = no interference and 10 = extreme interference, the perceived interference subscale assessed the degree to which fatigue in the past week interfered with life activities, concentration, relationships and quality of life. The total score for this subscale was obtained by averaging the items. Scores range from 0 to 10, with higher scores indicating greater fatigue interference. Reliability for the FSI-I in the present study was high (α = 0.94).

### 2.4. Analytic Strategy

All analyses were conducted using IBM SPSS Statistics (Version 22.0) (IBM, Chicago, IL, USA). Descriptive statistics were conducted to inspect variable distributions and assess the demographic and clinical characteristics of the sample. Based on our assumption of missing data completely at random (MCAR), listwise deletion was used throughout our analyses. We initially tested for associations among our predictor, mediator and outcome variables using a three-step approach [49]. First, the direct relationship between the predictor variable and the outcome variable is tested (Path C). If these two variables are determined to be significantly related, the relationship between the predictor variable and the hypothesized mediator variable is tested (Path A). Assuming significant relationships are observed, the final step is to test whether the direct relationship (Path C) between the predictor and outcome variable moves towards non-significance when the mediator is entered as a covariate (Path C’). Partial or full mediation is suggested with a decrease or complete loss of the C’ Path. Next, potential mediating factors were examined using a bootstrap method (PROCESS macro) [50]. Bootstrapping is often the preferred method to test for mediation because it does not violate assumptions of normality and allows for a more powerful test of mediation in small sample sizes [50]. The bootstrap method includes tests of each of the Baron and Kenny steps [49] of the relationships between predictor, mediator and outcome variables, as well as an examination of the indirect effects between predictor and outcome variables via hypothesized mediators.

Two longitudinal mediation models were assessed in the current study. We first tested whether depressive symptoms at 6-month follow-up mediated the relationship between baseline rejection issues and fatigue interference at 12-month follow-up. Here, multiple linear regression analyses were conducted to determine whether rejection issues at study entry predicted fatigue interference 12 months later (Path C) and whether rejection issues at study entry predicted depressive symptoms at the 6-month follow-up assessment (Path A). If these relationships were observed to be significant, Path C’ was examined using 6-month depressive symptoms as an additional covariate. Next, we assessed whether pain severity at 6-month follow-up mediated the relationship between baseline rejection issues and fatigue interference at 12-month follow-up using a similar statistical approach. Variables were determined to be mediators if all the relationships examined in the three-step process were significant, if the 95% confidence interval did not contain zero, and if the *p*-value of the indirect effects coefficient was less than 0.05 when calculated on the basis of 1000 bootstraps. All regression and bootstrapped analyses controlled for age, stage of cancer, type of procedure (lumpectomy or mastectomy), use of pain, sleep, anti-anxiety and anti-depressant medications, as well as intervention condition.

## 3. Results

### 3.1. Sample Characteristics

Participants were 240 women recently diagnosed with non-metastatic breast cancer. On average, women in the present study were 50.34 years old (*SD* = 9.03), had a mean income of $79,620 (*SD* = $67,079), and were partnered (150 women, 62.5% of the sample). Over half of the participants identified as Non-Hispanic White (152 women, 63.6% of the sample), while 61 identified as Hispanic/Latino (25.5% of the sample). Approximately 16 percent (N = 38) of the sample were diagnosed with stage 0 breast cancer (carcinoma in-situ). Women with stage I and II breast cancer comprised 37.8% (N = 90) and 38.2% (N = 91) of the current sample, respectively. A minority of women were diagnosed with stage III breast cancer (19 women, 8% of the sample). The majority of the sample had undergone a lumpectomy (122 women, 50.8%). On average, women underwent surgery 40.6 days (*SD* = 23.03) prior to the initial study assessment and had a BMI of 26.36 (*SD =* 5.59).

At study entry, women reported moderate fatigue interference (*M* = 3.63, *SD* = 1.97), mild pain severity (*M* = 2.28, *SD* = 1.62), and mild depressive symptoms (*M* = 7.52, *SD* = 5.46) [51,52,53]. Approximately 25% of women in the current sample reported taking medication to manage their pain at study entry. Baseline demographic characteristics of the sample are presented in Table 1. Descriptive statistics and internal consistency of main study variables at each assessment time point are reported in Table 2.

### 3.2. Post-Surgical Rejection Issues as a Predictor of Fatigue Interference at 12-Month Follow-Up

A multiple linear regression was conducted to determine if rejection issues at study entry (*M* = 3.70, *SD* = 1.73) predicted fatigue interference at the 12-month follow-up assessment (*M* = 2.52, *SD* = 1.63). Greater rejection issues at study entry significantly predicted greater fatigue interference at the 12-month follow-up assessment, above and beyond the effect of covariates and fatigue interference at study entry (*β* = 0.25, *t*(157) = 3.99, *SE* = 0.06, *p* < 0.01).

### 3.3. Testing the Mediating Role of Depressive Symptoms at 6-Month Follow-Up

First, Path A was examined using a multiple linear regression to determine if rejection issues at study entry predicted depressive symptoms at the 6-month follow-up assessment time point (*M* = 6.03, *SD* = 5.10). Adjusting for covariates and depressive symptoms at study-entry, rejection issues at study entry were not significantly associated with depressive symptomology at the 6-month follow-up time point (*β* = −0.02, *t*(170) = −0.09, *SE* = 0.18, *p* = 0.93). Therefore we could not test depressive symptoms as a mediator between post-surgical rejection issues and subsequent fatigue interference.

### 3.4. Testing the Mediating Role of Pain Severity at 6-Month Follow-Up

Path A was assessed using multiple linear regression to determine if rejection issues at study entry predicted pain severity at the 6-month follow-up time point (*M* = 1.90, *SD* = 1.58). Greater rejection issues at study entry were significantly associated with greater pain severity at the 6-month time point, above and beyond the effect of covariates, and pain severity at study entry (*β* = 0.19, *t*(162) = 3.56, *SE* = 0.05, *p* < 0.01). With evidence for significant Paths C and A, a regression analysis was conducted to examine the C’ Path. When the hypothesized mediator (i.e., pain severity at 6-month follow-up) was entered as an additional covariate, the regression of fatigue interference on pain severity remained significant (*β* = 0.32, *t*(143) = 3.16, *SE* = 0.10, *p* <0.01), while the relationship between baseline rejection issues and fatigue interference decreased in significance, suggesting partial mediation (*β* = 0.18, *t*(143) = 2.78, *SE* = 0.07, *p* < 0.01).

**Figure 1:**
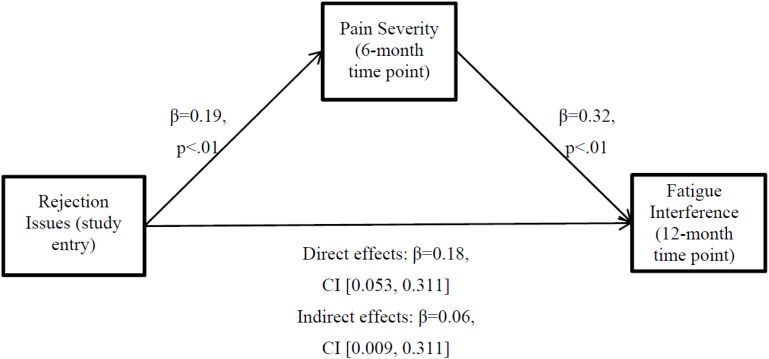
Path diagram of mediation model. Pain severity partially mediates the relationship between rejection issues and subsequent fatigue interference.

Accordingly, bootstrapping analyses with 1000 bootstrapped samples were used to determine indirect effects. Age, stage of cancer, intervention condition, procedure type, use of pain medications, anti-depressants, anti-anxiety medications, sleep medications as well as pain severity and fatigue interference at study-entry were used as covariates. The 95% confidence interval (CI) for the indirect effect estimate did not include zero (*β* = 0.06, 95% CI (0.009, 0.176)), indicating mediation at the 0.05 significance level. The effect size for this indirect effect was moderate (κ^2^ = 0.08, 95% CI (0.009, 0.241)). Observed direct effects were significant (*β* = 0.18, *SE* = 0.07, *t*(146) = 2.78, *p* < 0.01, 95% CI [0.053, 0.311]), further supporting partial mediation. As such, pain severity at 6-month follow-up may account for some, but not all of the relationship between baseline rejection issues and fatigue interference 12 months after study entry. Figure 1 presents the path diagram of this mediation model including path coefficients and significance levels.

## 4. Discussion

A cluster of symptoms including depression, pain, and fatigue is commonly observed and well-established in breast cancer [28,54]. There is a dearth of research examining the predictors of these symptoms, moreover, their associations across the trajectory of breast cancer treatment and survivorship. Extant literature has focused on low perceived social support and loneliness as potential antecedents to a treatment course troubled by poor emotional and physical well-being. Yet, the specific concerns responsible for perceived deficits in social support remain unclear. This study assessed the longitudinal relationships between rejection concerns in the period shortly after surgery and fatigue 12 months later. This interval characterizes the main timeline for primary treatment of breast cancer. Potential mediators of this relationship were examined based on mounting evidence for the relevance of intercurrent depressive symptoms and pain severity post-adjuvant therapy, approximately six months after breast cancer diagnosis.

We found that women who endorsed more rejection concerns in the weeks after surgery reported greater fatigue interference 12 months later. Mediation of this association via depressive symptoms was not supported in the current sample. We did find that greater rejection concerns at baseline predicted elevated pain levels six months later, and the relationship between rejection concerns at study entry and fatigue interference at 12 months was partially mediated by pain severity at the six-month time point. This suggests that the more women are concerned about rejection in the weeks after breast cancer surgery the more they may be likely to experience more fatigue interference during survivorship, possibly as a result of elevated pain levels during their treatment course.

Our findings are largely consistent with existing literature on the psychological and physical symptom trajectory across breast cancer diagnosis, treatment, and survivorship. There is much support for the relationship between low perceived social support and cancer-related fatigue, yet the current study is one of a few to highlight the possible development of fatigue over time in association with feared social rejection prior to adjuvant treatment [28,31,55]. To our knowledge, this is the first study to assess fear of rejection as a predictor of subsequent post-adjuvant therapy fatigue interference.

Contrary to current literature, the present study did not observe a relationship between baseline rejection concerns and depressive symptoms after completion of adjuvant therapy [29]. Furthermore, there was no support for mediation between rejection concerns at study entry and fatigue interference due to depressive symptomatology after treatment. Depressive symptoms in the current sample were measured using the Hamilton Rating Scale for Depression, with mean values ranging from mild to no depressive symptoms [52]. It is possible that these observed levels of depressive symptomatology were simply too low to influence subsequent fatigue interference during survivorship. Moreover, because we excluded women presenting with Major Depressive Disorder and other forms of significant psychopathology it is possible that we sampled a group of women who were less prone to express difficulties with depression.

We did find support for partial mediation between rejection concerns and subsequent fatigue interference via pain severity after completion of adjuvant therapy. It is plausible that women who experience greater rejection concerns also have lower perceived social support, and their rumination surrounding these concerns makes it more difficult for them to effectively manage their pain due to increased self-focus and pre-occupation with bodily states. As a result, levels of pain may increase, which could ultimately lead to poorer sleep and greater interference with daily functioning due to fatigue [56]. Future research should investigate sleep quality as an additional mediator of the relationship between social rejection concerns and subsequent fatigue interference.

### 4.1. Strengths and Limitations

The current study has many strengths including a large sample size and a longitudinal design. Mediation models were tested using three assessment time points (post-surgery, six months post-baseline, 12 months post-baseline), allowing us to fully test a longitudinal model. The current study is one of a few research studies to elucidate a longitudinal mediation pathway between commonly observed symptoms of breast cancer treatment and survivorship (e.g., pain and fatigue) [54,56]. The use of bootstrapping methods in the mediation analyses is another strength of this study as it does not rely on assumptions of normality and is a more powerful test than other commonly used mediation approaches [50].

Psychosocial and physical symptoms experienced by women with breast cancer have complex relationships with one another that need to be considered in symptom management research and clinical practice. The findings from this study provide additional support for a potential cascade of symptoms (pain, and fatigue) throughout breast cancer treatment and survivorship that may be initiated by rejection concerns or social isolation [28,31,57,58].

One limitation of this study is the reliance on the HRSD as a measure of depression. Since the HRSD is administered by an interviewer, it may be particularly prone to social desirability bias, such that women may under-report their depressive symptoms. A second limitation relates to the use of the FSI-I subscale. As discussed previously, this is a measure designed to assess the degree to which fatigue interferes with daily life activities. Use of a multidimensional measure of fatigue may lead to more generalizable findings. A third limitation involves the generalizability of our findings to other groups of breast cancer patients and survivors. It is unclear if similar findings would occur if different time points in the treatment trajectory were used, as the issues women face are constantly changing as they move through treatment and into survivorship [59]. These results also may not generalize to women who have metastatic (Stage IV) breast cancer as our sample is entirely composed of women with non-metastatic breast cancer. Although one third of our sample identified as an ethnic minority (Hispanic, African-American, or Asian), women in this sample were predominantly Non-Hispanic White, partnered, well-educated, and of a high income level which further limits our ability to apply these findings to more diverse populations. The participants’ relatively low baseline scores on symptoms of depression, pain severity, and fatigue further limit generalizability. Further work is needed to determine if these relationships would hold in samples experiencing clinical levels of these symptoms. Lastly, the use of listwise deletion is a limitation as it may influence analytic procedures due to missing data over time.

### 4.2. Future Work and Clinical Implications

Future studies are needed to clarify these findings. If pain severity after completion of adjuvant therapy for breast cancer only partly explains the association between fear of rejection at time of diagnosis and fatigue interference 12 months later, there is a need to determine what else is responsible for this relationship. It is conceivable that the relationship between rejection concerns, pain, and fatigue interference is also affected by sleep quality [56]. Anxiety about the future and intrusive thoughts pertaining to breast cancer treatment and its side effects are commonly observed after adjuvant therapy [60,61,62] and may help explain the relationship between fear of rejection, pain, and fatigue interference, since some research has reported breast cancer-related anxiety to be associated with fatigue [26,63]. Lastly, the role of coping strategies is likely relevant when considering the relationship between pain severity and subsequent fatigue interference [64,65]. It is plausible that certain coping techniques to deal with treatment-related pain may affect the presence and severity of fatigue interference, such that adaptive coping (e.g., positive reappraisal) may lessen fatigue interference whereas maladaptive coping (e.g., pain catastrophizing) may exacerbate fatigue interference [64,65,66,67,68]. In sum, sleep problems, anxiety and catastrophizing surrounding pain may place an extra strain on energy levels, above and beyond the lingering influence of adjuvant therapy, such that these women experience surplus levels of fatigue-related interference in their lives well into survivorship.

Although our aims were exploratory in nature, our findings have important clinical implications for women recently diagnosed with non-metastatic breast cancer. Findings suggest that if psychosocial interventions target women’s fear of social rejection and interpersonal skills training (e.g., assertiveness) early in treatment, reductions in physical symptom burden during treatment (i.e., pain severity) and into survivorship (i.e., fatigue interference) may follow. Group based interventions may be particularly helpful in reducing fears of social rejection. Future work should examine intervention effects on fear of social rejection, pain severity and fatigue interference at different points in treatment to improve symptom management during the entire trajectory of breast cancer treatment, and survivorship.

## 5. Conclusions

The present findings clarify temporal associations between post-surgical psychological status and a significant quality of life issues (e.g., pain severity, and fatigue interference) during survivorship. Women who endorsed more rejection concerns in the weeks after surgery reported greater fatigue interference 12 months later. Greater rejection concerns at baseline predicted greater pain severity six months later, and levels of pain severity partially mediated the relationship between rejection concerns and fatigue interference. Future work is needed to replicate these findings in diverse populations of breast cancer survivors, as well as establish other mediators of the observed relationship between rejection concerns and fatigue interference. Future studies should also investigate whether psychosocial interventions that aim to reduce fear of rejection additionally reduce physical symptom burden.

## Figures and Tables

**Table 1:** Demographic and medical variables collected at study-entry. N = 240 unless otherwise reported.

**Participant Characteristic**	**Mean**	***SD***
Age at diagnosis	50.34	9.03
Positive Lymph Nodes (N *=* 239*)*	1.56	3.32
Days since surgery	40.64	23.03
Income, in thousands (N *=* 213*)*	79.62	67.08
Years of education	15.58	2.38
**Stage of disease**	**N**	**Percentage**
Stage *		
0	38	15.8
I	90	37.5
II	91	37.9
III	19	7.9
Type of surgery *		
Lumpectomy	122	50.8
Mastectomy	118	49.2
**Menopausal Status**		
Pre	107	44.6
Peri/Post	133	55.4
Radiation *	134	55.8
Chemotherapy *	127	52.9
Hormonal Therapy *	161	67.1
**Ethnicity**		
Non-Hispanic White	152	63.3
Hispanic	61	25.4
African American	21	8.8
Asian	5	2.1
Partnered	150	62.5
Employed	178	74.2

* confirmed by chart review after study completion. Note. Percentages that do not equal 100% are due to occasional missing data.

**Table 2:** Descriptive statistics and internal consistency of key study variables at each assessment time point.

Study Variable	T1	T2	T3
Fatigue Interference			
N	226 †	189	190
*M*	3.63	2.88	2.53
*SD*	1.97	1.77	1.63
α	0.9	0.9	0.9
Pain Severity			
N	226 †	189	190
*M*	2.28	1.90	1.81
*SD*	1.62	1.58	1.54
α	0.9	0.9	0.9
Rejection Issues			
N	229 †	184	184
*M*	3.70	3.46	3.40
*SD*	1.73	1.23	0.99
α	0.8	0.6	0.4
Depressive Symptoms			
N	231 †	195	180
*M*	7.52	6.03	5.96
*SD*	5.46	5.10	5.02
α	0.8	0.8	0.8
Pain Medication			
% (N) Yes	25.0 (60)	10.0 (24)	10.0 (24)
% (N) No	75.0 (180)	70.4 (169)	69.2 (166)
Anti-Depressants			
% (N) Yes *	10.8 (26)	9.6 (23)	12.1 (29)
% (N) No	89.2 (214)	70.8 (170)	67.1 (161)
Sleep Medication			
% (N) Yes	17.9 (43)	9.2 (22)	11.7 (28)
% (N) No	82.1 (197)	70.8 (170)	67.9 (163)
Anti-Anxiety Medication			
% (N) Yes	17.5 (42)	12.1 (29)	14.2 (34)
% (N) No	82.5 (198)	68.3 (164)	65.0 (156)

* unspecified as to whether participants were on anti-depressants for depression or to manage treatment side effects such as hot flashes; † Although N = 240 were enrolled and completed baseline assessments, smaller sample size for these measures at baseline are due to missing data on one or more study variables and listwise deletion. Note: Percentages that do not equal 100% are due to occasional missing data.
